# Risk Stratification Model for Predicting the Overall Survival of Elderly Triple-Negative Breast Cancer Patients: A Population-Based Study

**DOI:** 10.3389/fmed.2021.705515

**Published:** 2021-09-21

**Authors:** Xiaozhu Liu, Song Yue, Haodong Huang, Minjie Duan, Binyi Zhao, Jin Liu, Tianyu Xiang

**Affiliations:** ^1^Department of Cardiology, The Second Affiliated Hospital of Chongqing Medical University, Chongqing, China; ^2^Department of Gynecology and Obstetrics, The Second Affiliated Hospital of Chongqing Medical University, Chongqing, China; ^3^College of Medical Informatics, The Chongqing Medical University, Chongqing, China; ^4^Department of Personnel, Science and Education, The Third Affiliated Hospital of Chongqing Medical University, Chongqing, China; ^5^Information Center, The University-Town Hospital of Chongqing Medical University, Chongqing, China

**Keywords:** triple-negative breast cancer, risk stratification, adjuvant radiotherapy, prediction model, web app

## Abstract

**Background:** The objective of this study was to evaluate the prognostic value of clinical characteristics in elderly patients with triple-negative breast cancer (TNBC).

**Methods:** The cohort was selected from the Surveillance, Epidemiology, and End Results (SEER) program dating from 2010 to 2015. Univariate and multivariate analyses were performed using a Cox proportional risk regression model, and a nomogram was constructed to predict the 1-, 3-, and 5-year prognoses of elderly patients with TNBC. A concordance index (C-index), calibration curve, and decision curve analysis (DCA) were used to verify the nomogram.

**Results:** The results of the study identified a total of 5,677 patients who were randomly divided 6:4 into a training set (*n* = 3,422) and a validation set (*n* = 2,255). The multivariate analysis showed that age, race, grade, TN stage, chemotherapy status, radiotherapy status, and tumor size at diagnosis were independent factors affecting the prognosis of elderly patients with TNBC. Together, the 1 -, 3 -, and 5-year nomograms were made up of 8 variables. For the verification of these results, the C-index of the training set and validation set were 0.757 (95% CI 0.743–0.772) and 0.750 (95% CI 0.742–0.768), respectively. The calibration curve also showed that the actual observation of overall survival (OS) was in good agreement with the prediction of the nomograms. Additionally, the DCA showed that the nomogram had good clinical application value. According to the score of each patient, the risk stratification system of elderly patients with TNBC was further established by perfectly dividing these patients into three groups, namely, low risk, medium risk, and high risk, in all queues. In addition, the results showed that radiotherapy could improve prognosis in the low-risk group (*P* = 0.00056), but had no significant effect in the medium-risk (*P* < 0.4) and high-risk groups (*P* < 0.71). An online web app was built based on the proposed nomogram for convenient clinical use.

**Conclusion:** This study was the first to construct a nomogram and risk stratification system for elderly patients with TNBC. The well-established nomogram and the important findings from our study could guide follow-up management strategies for elderly patients with TNBC and help clinicians improve individual treatment.

## Introduction

Breast cancer (BC) is one of the most common cancers in women and the leading cause of death from malignancies. According to the latest global cancer data released by the International Agency for Research on Cancer (IARC) in 2020, BC has become the most diagnosed cancer around the world, with its incidence rate increasing every year (1, 2). Triple-negative BC (TNBC) is a subtype of BC, wherein the estrogen receptor (ER), progesterone receptor (PR), and human epidermal growth factor receptor 2 (HER2) are negatively expressed (3, 4). Triple-negative BC also has many gene expression subtypes, such as basal-like 1, basal-like 2, immunomodulatory, mesenchymal (M), mesenchymal stem-like, and luminal androgen receptors, with the most common subtype being basal-like (5). Furthermore, the proportion of newly diagnosed patients with *in situ* or invasive BC over the age of 70 is expected to increase from 24.3 to 34.8% by 2030 (1). Of these cases, elderly women are most often diagnosed with estrogen-derived tumors, which can be treated with targeted hormone therapy with good prognoses. However, patients with the triple-negative subtype of BC, which is insensitive to targeted hormone therapy, account for 15–20% of all BC cases (6). Triple-negative BC also has a poorer prognosis and higher death rate and invasiveness compared with other BC subtypes (7). Although the number of elderly patients with this disease is increasing, few clinical trials and studies have been conducted in this age group. The treatment of older patients with BC is compounded by issues with functional status assessment, comorbidities, life expectancy, and tolerability. Adjuvant therapy, e.g., radiotherapy, can improve the prognosis of BC patients. However, the value of TNBC adjuvant radiotherapy is still controversial, especially in elderly patients (8).

Because patients with TNBC have substantial limitations in the choice of treatment modality, the early prediction of overall survival (OS) may improve patient outcomes. Therefore, a risk stratification tool for predicting the OS of TNBC is needed. Network-based stratification has previously been studied to predict survival in cancer patients, but this model was mainly based on genetic data which is costly to collect and has some limitations (9).

The tumor, nodes, and metastases (TNM) staging system is a tool used for predicting the prognoses of cancer patients by calculating their clinical stage according to the guidelines of the American Joint Committee on Cancer (AJCC) based on tumor size or location (T), regional lymph node metastasis (N), and distant metastasis (M). However, the current TNM staging system may not be sufficient to cover tumor biology and predict all BC outcomes, especially for treatment decisions in patients with TNBC (10). Some oncology studies have shown that the nomogram has an advantage over the TNM staging system in the study of tumor prognosis (11, 12). The nomogram is a handy tool for predicting and quantifying the likelihood of a patient to experience a specific clinical event. Moreover, this tool may be valuable for clinical decision-making in risk stratification, personalized treatment, and clinical trial design.

Most existing TNBC models were developed based on patients younger than 70 years of age; however, predictive models specifically designed for patients older than 70 years of age are still lacking. The purpose of this study was to construct and validate a new predictive model for predicting TNBC outcomes in elderly patients using cohort data from the Surveillance, Epidemiology, and End Results (SEER) database. Established in 1973, the SEER database aims to reduce the cancer burden in the US population. It also contains data on the incidence, mortality, and prevalence of more than a million patients with cancer, covering approximately 28% of the US population, so the data are representative (13).

## Materials and Methods

### Patient Sources and Screening Criteria

The current data were from the SEER database. The data of patients with TNBC from 2010 to 2015 were screened from the SEER database using the latest SEER^*^STAT version 8.3.8 (National Cancer Institute, https://seer.cancer.gov/). The most common histological codes for TNBC were also included to rule out the potential confounding of rare histology, namely, invasive ductal (ICD-O-3 8500/3) and invasive lobular carcinomas (ICD-O-3 8520/3). The following inclusion and exclusion criteria were used for screening:

Inclusion criteria: (1) women aged 70 and above; (2) a positive histological diagnosis of a unilateral BC without an autopsy or death certificate; (3) a negative ER/PR/HER2; (4) a positive follow-up; (5) being AJCC stage I–III and having a histological grade I–III; (6) infiltrating ductal (IDC) and infiltrating lobular carcinomas (ILC).

Exclusion criteria: men with BC that were non-invasive and M1 patients; patients with incomplete clinicopathological information such as ER, PR, HER2, tumor grade, and TN stage; incomplete or unclear data on other indicators.

### Study Variables and Outcomes

The study analyzed 12 indices from the selected characteristics of patients, including age at diagnosis (70–74, 75–79, 80–84, and more than 84 years of age), race (black, white, others including American Indian/Alaska Native and Asian/Pacific Islander), grade (I–III), AJCC stage (I–III), T stage (T1–T4), N stage (N0–N3), laterality (left or right), histological subtype (IDC, ILC), marital status, tumor size, radiotherapy, and chemotherapy conditions. Unmarried refers to patients who are divorced, separated, single, or widowed. Grade I stands for well-differentiated, grade II stands for moderately differentiated, and grade III stands for poorly differentiated. The tumor sizes (≤ 5, 6–10, 11–20, 21–50, and >50 mm) were converted into classification variables to satisfy the linear hypothesis. The primary outcome of the study was the OS rate, which was defined as death associated with any cause from the date of diagnosis until the last follow-up outcome, i.e., survival or death. All data in the SEER database were free, and TNM staging was based on the seventh edition of the AJCC clinical staging criteria.

### Statistical Analysis

All eligible cases were randomly divided into either the training or validation cohort (the split ratio was 6:4), and. The training cohort was used to construct the nomogram and establish the predictive model and risk stratification system. The data of the validation cohort were used to carry out the validation of the model.

The corresponding 95% CIs and hazard ratios (HRs) for every potential prognostic variable were established by univariate and multivariate Cox proportional hazards regression models in a forward stepwise manner. The significant variables in the univariate analyses (*P* < 0.05) were included in the multivariate analyses. The software SPSS 24 (SPSS, Chicago, IL) was used for these statistical analyses. Afterward, the created nomogram could provide visualized risk predictions using the RMS packages and the survival packages of R 4.0.2 (CRAN project, Lucent Technologies, New Jersey, USA. www.r-project.org) based on the results of these multivariable analyses (14). The accuracy of the nomogram was then assessed by discrimination and calibration evaluation. Discrimination, which means the ability of a model to distinguish patients with different outcomes, was evaluated using the concordance index (C-index) as the measuring tool. On the other hand, calibration curves (1,000 bootstrap resamples) were used to test the calibration of the nomogram. Furthermore, calibration plots for 1-, 3-, and 5-year OS were carried out in the training cohort and validation cohort. A decision curve analysis (DCA) was also used to evaluate the TNM staging system and the clinical net benefits of the predictive model (15).

In addition, a risk stratification system was established based on the total score of each patient obtained from the nomogram. Afterward, the X-Tile software (Robert L. Camp, Yale University, New Haven, Connecticut, USA) was used to evaluate the optimal cutoff value of the total score of each patient. These values were then used to divide the patients into three prognostic groups, namely, low risk, medium risk, and high risk (16). Kaplan–Meier curves and the log-rank test were also used to illustrate and compare the OS of patients in different risk groups.

## Results

### Patient Characteristics

**Figure 1:**
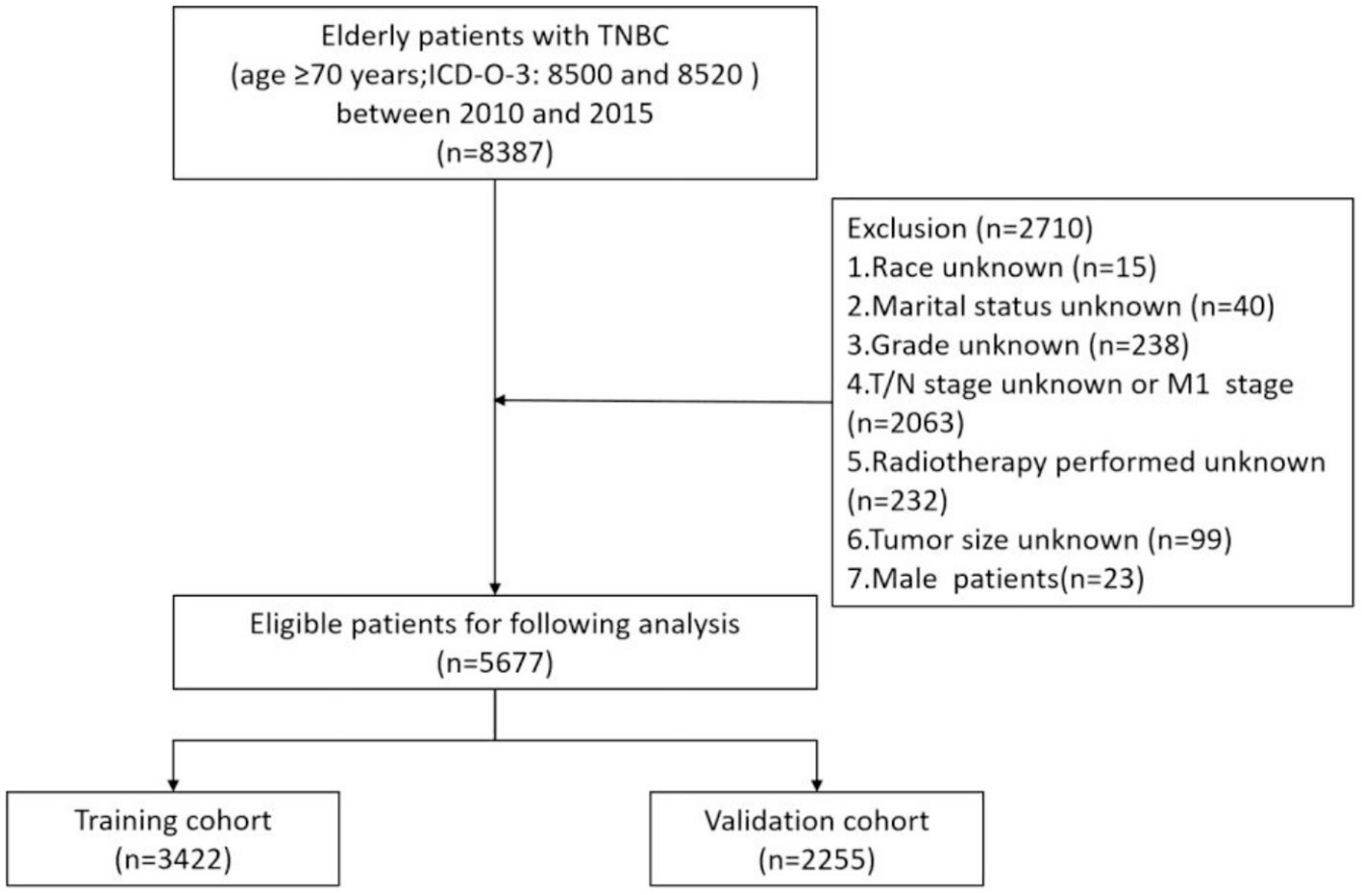
Patient selection flowchart.

The flowchart of the patient selection process is shown in Figure 1. A total of 5,677 eligible patients were identified from the SEER database between 2010 and 2015. The clinicopathological characteristics and treatment status of all these patients (see Table 1), including 3,422 patients in the training set and 2,255 patients in the validation set, showed no statistical difference between the two data sets (*P* > 0.05). Among all the patients, 2,193 (38.6%) were 70–74 years old and 1,493 (26.3%) were 75–79 years old. In addition, 53.36 (3,029 out of 5,677), 36.18 (2,054 out of 5,677), 5.8 (329 out of 5,677), and 4.67% (265 out of 5,677) of the patients had stage T1, T2, T3, and T4 tumors, respectively. Furthermore, 27.4% (1,548 out of 5,677) of the patients had negative *N* stages, while 72.7% (4,129 out of 5,677) had positive *N* stages. Depending on the treatment modality, the treatment rates of radiotherapy and chemotherapy for patients were 44.5 (2,524 out of 5,677) and 41.3% (2,343 out of 5,677), respectively. The median follow-up time in the study cohort was 46 months [interquartile range (IQR) 28–64, 95% CI 44.897–47.103]. The 1-, 3-, and 5-year OS rates in the training set were 91.1 (90.6–91.6%), 74.2 (73.4–75%), and 61.8% (60.7–62.9%), respectively, while the 1-, 3-, and 5-year OS rates in the validation set were 91 (90.4–91.6%), 72.4 (71.4–73.4%), and 61.4% (60.1–62.7%), respectively.

**Table 1:** Clinicopathological characteristics of patients with triple-negative breast cancer (TNBC) in the present study.

**Characteristics**	**All** ** patients**	**Training** ** cohort**	**Validation** ** cohort**	
	***N* = 5,677**	***N* = 3,422**	***N* = 2,255**	***p***
**Age, year**				0.895
70–74	2,193 (38.6%)	1,319 (38.5%)	874 (38.8%)	
75–79	1,493 (26.3%)	904 (26.4%)	589 (26.1%)	
80–84	1,091 (19.2%)	665 (19.4%)	426 (18.9%)	
>84	900 (15.9%)	534 (15.6%)	366 (16.2%)	
**Race**				0.59
White	4,488 (79.1%)	2,699 (78.9%)	1,789 (79.3%)	
Black	845 (14.9%)	521 (15.2%)	324 (14.4%)	
Other^a^	344 (6.0%)	202 (5.9%)	142 (6.3%)	
**Grade**				0.121
I	164 (2.9%)	87 (2.6%)	77 (3.4%)	
II	1,333 (23.5%)	795 (23.2%)	538 (23.9%)	
III	4,180 (73.6%)	2,540 (74.2%)	1,640 (72.7%)	
**T**				0.961
T1	3,029 (53.36%)	1,832 (53.54%)	1,197 (53.08%)	
T2	2,054 (36.18%)	1,231 (35.97%)	823 (36.50%)	
T3	329 (5.80%)	201 (5.87%)	128 (5.68%)	
T4	265 (4.67%)	158 (4.62%)	107 (4.75%)	
***N***				0.997
N0	4,129 (72.73%)	2,488 (72.71%)	1,641 (72.77%)	
N1	1,020 (17.98%)	615 (17.97%)	405 (17.96%)	
N2	318 (5.60%)	191 (5.58%)	127 (5.63%)	
N3	210 (3.70%)	128 (3.74%)	82 (3.64%)	
**Laterality**				0.252
Left	2,952 (52.0%)	1,801 (52.6%)	1,151 (51.0%)	
Right	2,725 (48.0%)	1,621 (47.4%)	1,104 (49.0%)	
**Histology**				0.769
IDC	5,534 (97.5%)	3,338 (97.5%)	2,196 (97.4%)	
ILC	143 (2.5%)	84 (2.5%)	59 (2.6%)	
**Marriage**				1
No	3,257 (57.4%)	1,963 (57.4%)	1,294 (57.4%)	
Yes	2,420 (42.6%)	1,459 (42.6%)	961 (42.6%)	
**AJCC**				0.671
I	2,698 (47.5%)	1,612 (47.1%)	1,086 (48.2%)	
II	2,168 (38.2%)	1,312 (38.3%)	856 (38.0%)	
III	811 (14.3%)	498 (14.6%)	313 (13.9%)	
**Chemotherapy**				0.964
No/Unknown	3,334 (58.7%)	2,011 (58.8%)	1,323 (58.7%)	
Yes	2,343 (41.3%)	1,411 (41.2%)	932 (41.3%)	
**Radiotherapy**				1
No	3,153 (55.5%)	1,901 (55.6%)	1,252 (55.5%)	
Yes	2,524 (44.5%)	1,521 (44.4%)	1,003 (44.5%)	
**Tumor size, mm**				0.563
≤ 5	360 (6.3%)	208 (6.1%)	152 (6.7%)	
6–10	827 (14.6%)	492 (14.4%)	335 (14.9%)	
11–20	1,870 (32.9%)	1,150 (33.6%)	720 (31.9%)	
21–50	2,157 (38.0%)	1,287 (37.6%)	870 (38.6%)	
>50	463 (8.2%)	285 (8.3%)	178 (7.9%)	

a *Others, American Indian, Alaska Native, Asian, and Pacific Islander; IDC, infiltrating duct carcinoma; ILC, infiltrating lobular carcinoma*.

### Univariate and Multivariate Analyses

In the training set, univariate Cox regression analyses were performed to determine clinical features with *P* < 0.05, including age at diagnosis, race, pathological grade, TN stage, marital status, chemotherapy status, radiotherapy status, and tumor size. These features were then analyzed in a multivariate Cox regression model (Table 2), which showed that the clinical features associated with survival included age (70–74 as a reference; 75–79: HR 1.264, 95% CI 1.057–1.511; 80–84: HR 1.608, 95% CI 1.335–1.937; >84: HR 2.142, 95% CI 1.767–2.597), race (white as a reference; black: HR 1.197, 95% CI 1.013–1.414; others: HR 0.697, 95% CI 0.525–0.924), tumor grade (grade I as a reference; grade III: HR 1.829, 95% CI 1.021–3.278), T stage (T1 as a reference; T4: HR 2.279, 95% CI 1.153–4.503), N stage (N0 as a reference; N1: HR 1.702, 95% CI 1.456–1.989; N2: HR 2.391, 95% CI 1.901–3.008; N3: HR 3.175, 95% CI 2.491–4.048), chemotherapy (no chemotherapy as a reference; chemotherapy: HR 0.625, 95% CI 0.537–0.727), radiotherapy (no radiotherapy as a reference; radiotherapy: HR 0.573, 95% CI 0.499–0.658), and tumor size (≤ 5 as a reference; 11–20: HR 2.147, 95% CI 1.384–3.333; 21–50: HR 3.165, 95% CI 1.339–7.479; > 50: HR 2.501, 95% CI 1.085–5.764). Finally, these clinical predictive features were included in the established nomogram for further analyses.

**Table 2:** Univariate and multivariate analyses of overall survival (OS) in the current cohort.

**Variable**	**Univariate analysis**		**Multivariate analysis**	
	**HR (95% CI)**	***P***	**HR (95% CI)**	***P***
**Age**				
70–74	Reference		Reference	
75–79	1.375 (1.154, 1.639)	<0.001	1.264 (1.057, 1.511)	0.01
80–84	2.032 (1.706, 2.422)	<0.001	1.608 (1.335, 1.937)	<0.001
>84	3.452 (2.914, 4.088)	<0.001	2.142 (1.767, 2.597)	<0.001
**Race**				
White	Reference		Reference	
Black	1.216 (1.033, 1.432)	0.019	1.197 (1.013, 1.414)	0.035
Other	0.850 (0.642, 1.125)	0.255	0.697 (0.525, 0.924)	0.012
**Grade**				
I	Reference		Reference	
II	1.913 (1.068, 3.424)	0.029	1.504 (0.83, 2.727)	0.178
III	2.588 (1.463, 4.576)	0.001	1.829 (1.021, 3.278)	0.042
**T**				
T1	Reference		Reference	
T2	2.423 (2.106, 2.788)	<0.001	1.161 (0.545, 2.471)	0.699
T3	4.485 (3.618, 5.56)	<0.001	2.03 (0.966, 4.267)	0.062
T4	5.832 (4.667, 7.288)	<0.001	2.279 (1.153, 4.503)	0.018
**N**				
N0	Reference		Reference	
N1	2.146 (1.853, 2.486)	<0.001	1.702 (1.456, 1.989)	<0.001
N2	3.003 (2.423, 3.723)	<0.001	2.391 (1.901, 3.008)	<0.001
N3	4.553 (3.638, 5.698)	<0.001	3.175 (2.491, 4.048)	<0.001
**Laterality**				
Left	Reference			
Right	1.036 (0.916, 1.171)	0.577		
**Histology**				
IDC	Reference			
ILC	1.207 (0.839, 1.736)	0.31		
**Marriage**				
No	Reference		Reference	
Yes	0.669 (0.588, 0.761)	<0.001	0.981 (0.856, 1.125)	0.788
**AJCC**				
I	Reference			
II	1.041 (0.91, 1.19)	0.558		
III	1.069 (0.89, 1.284)	0.475		
**Chemotherapy**				
No/Unknown	Reference		Reference	
Yes	0.644 (0.565, 0.736)	<0.001	0.625 (0.537, 0.727)	<0.001
**Radiotherapy**				
No	Reference		Reference	
Yes	0.488 (0.427, 0.558)	<0.001	0.573 (0.499, 0.658)	<0.001
**Tumor size**				
≤ 5	Reference		Reference	
6–10	1.656 (1.034, 2.651)	0.036	1.597 (0.995, 2.563)	0.052
11–20	2.3 (1.487, 3.558)	<0.001	2.147 (1.384, 3.333)	0.001
21–50	4.88 (3.184, 7.481)	<0.001	3.165 (1.339, 7.479)	0.009
>50	9.541 (6.113, 14.891)	<0.001	2.501 (1.085, 5.764)	0.031

*Others, American Indian, Alaska Native, Asian, and Pacific Islander; IDC, infiltrating duct carcinoma; ILC, infiltrating lobular carcinoma; HR, hazard ratio; 95% CI, 95% confidence index*.

### Nomogram Development and Validation

**Figure 2:**
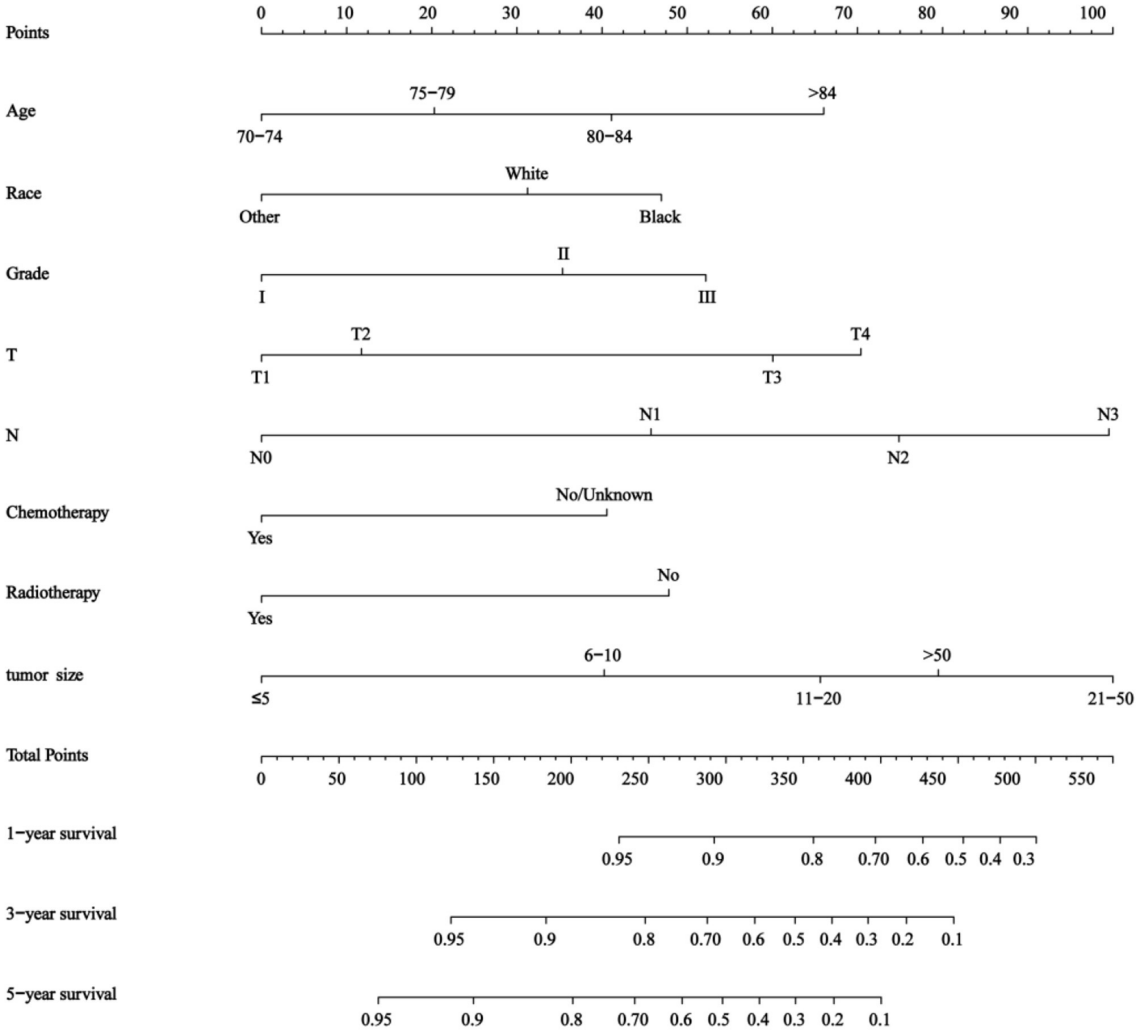
Nomogram for predicting 1-, 3-, and 5- year overall survival (OS) in elderly patients with triple-negative breast cancer (TNBC).

The results of this study identified eight independent predictive features based on the multivariate Cox regression and constructed a predictive nomogram (Figure 2), including age, race, tumor grade, T stage, N stage, chemotherapy status, radiotherapy status, and tumor size. Scores were assigned to each clinical feature, and the estimated 1-, 3-, and 5-year OS probabilities were easily obtained by adding up all the scores for the eight clinical features and drawing a vertical line between the total score and the survival probability axis.

The nomogram showed that tumor size and *N* stage had a significant influence on prognosis, followed by T stage, age, tumor grade, race, radiotherapy status, and chemotherapy status. The C-indices of the training and validation sets were 0.757 (95% CI 0.743–0.772) and 0.75 (95% CI 0.742–0.768), respectively.

**Figure 3:**
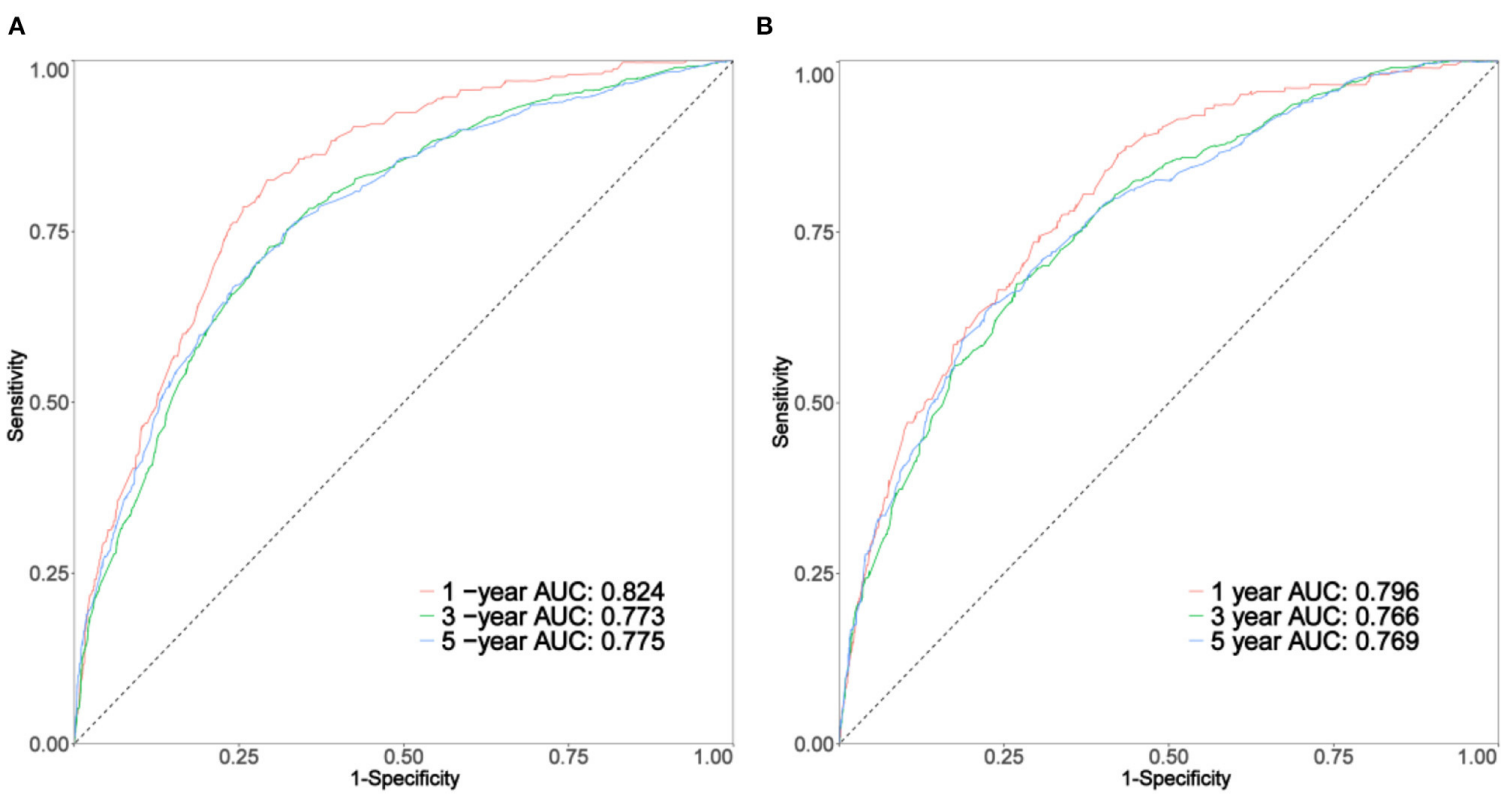
The curve of **(A,B)** represents the receiver operating characteristic (ROC) of 1, 3, and 5 years of the training set and validation set, respectively.

**Figure 4:**
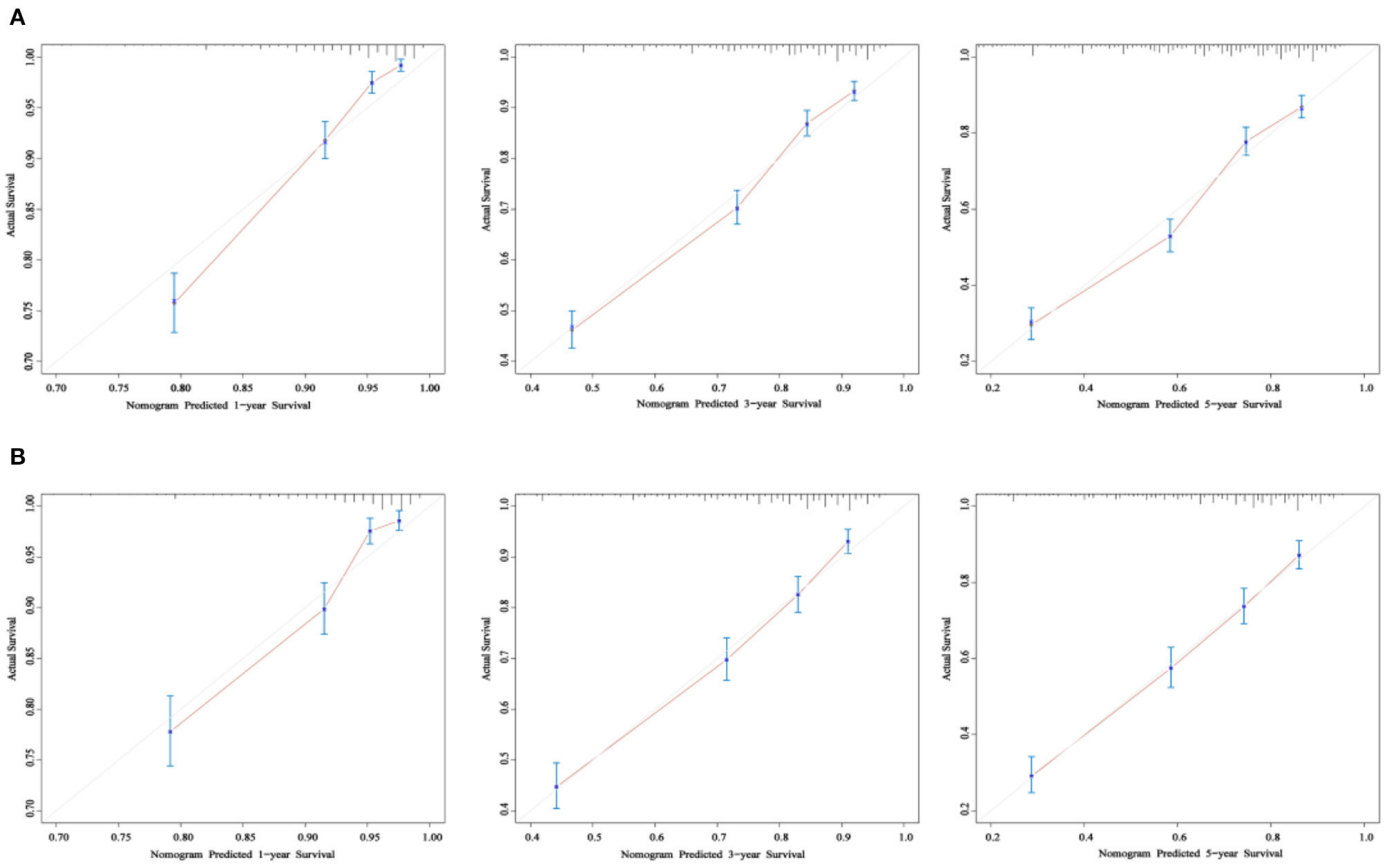
The calibration curve of OS at 1-, 3-, and 5- years for the training cohort **(A)** and the validation cohort **(B)**. The x-axis refers to the probability of survival and the y-axis means actual survival. The gray lines represent the perfect calibration models in which the predicted probabilities are identical to the actual probabilities.

**Figure 5:**
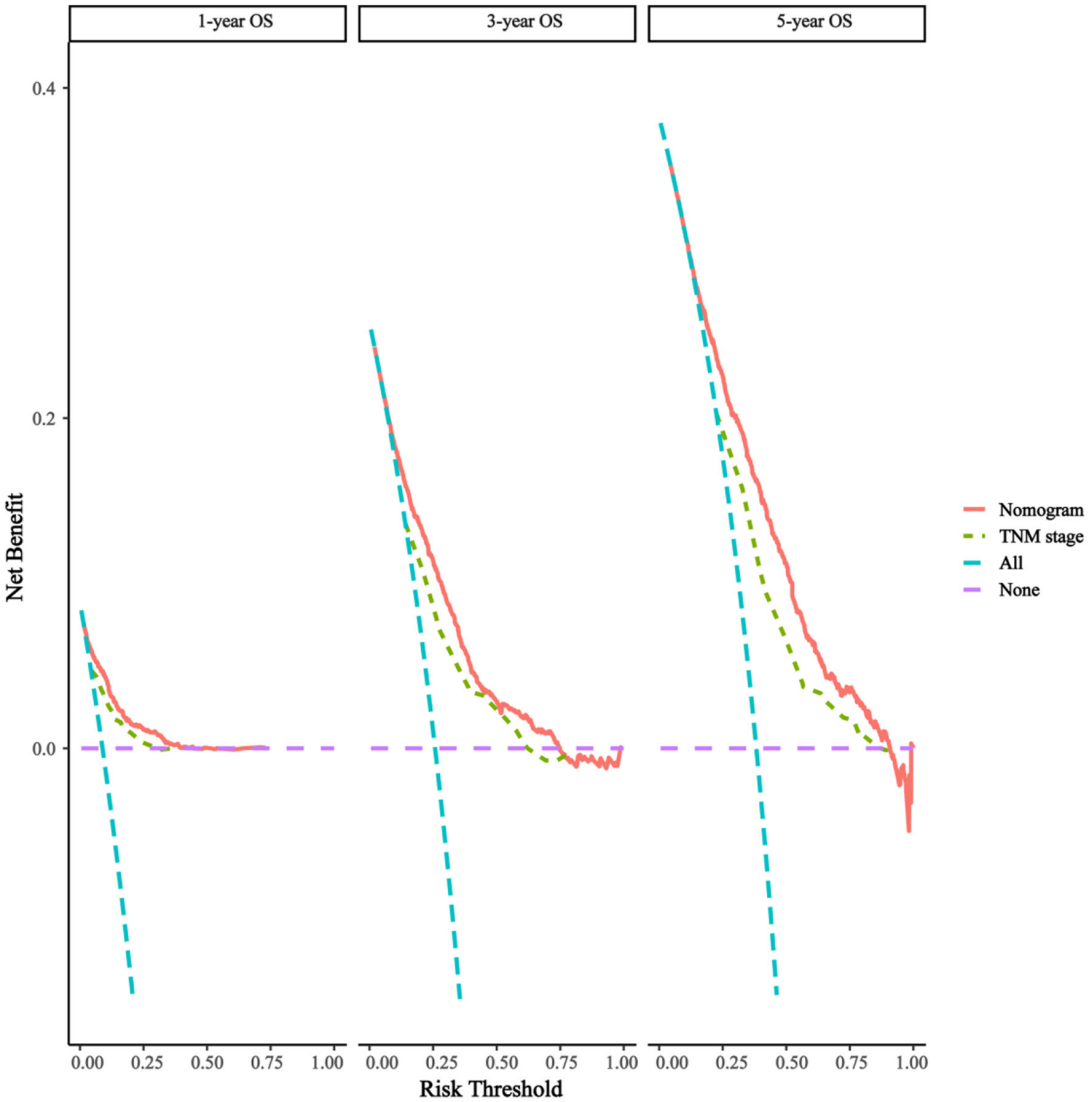
Decision curves of the nomogram predicting OS. The x-axis represents the threshold probabilities and the y-axis measures the net benefit, which is calculated by adding the true positives and subtracting the false positives.

As shown in Figures 3A,B, the 1-, 3-, and 5-year areas under the curve (AUCs) of the training set were 0.824, 0.773, and 0.775, respectively. On the other hand, the 1-, 3-, and 5-year AUCs of the validation set were 0.796, 0.766, and 0.769, respectively. The results all showed that the prediction accuracy of the model was high. The calibration diagram of the training and validation sets adopted 1,000 bootstraps, indicating a good consistency between the predicted results and the actual results (Figures 4A,B). We also compared the DCA curve between the training set and the TNM staging system to determine the clinical practicability of the nomogram. The results showed that, compared with the TNM staging system, the nomogram had a better clinical net benefit and a larger threshold probability range in predicting 1-, 3-, and 5-year OS in patients with TNBC (Figure 5).

### Risk Stratification Analysis

**Figure 6:**
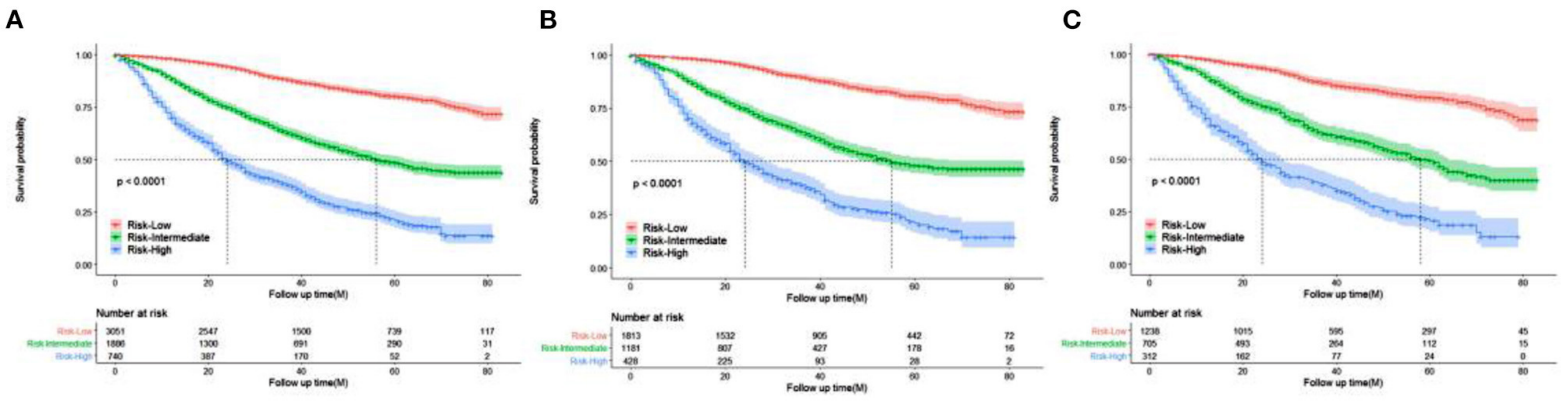
Kaplan–Meier curves of OS for patients in the low-, intermediate-, and high-risk groups. **(A)** all cohorts **(B)** training cohort **(C)** validation cohort.

Patients were divided into three prognostic groups according to the optimal cutoff value: low-risk group (3,051 out of 5,677, 53.74%, score 40.6–256), medium-risk group (188 out of 5,677, 33.22%, score 256–345.5), and high-risk group (740 out of 5,677, 13.04%, score 345.5–523.5) (Figures 6A–C). The Kaplan–Meier curve in the low-risk group showed that the risk stratification system could accurately distinguish the OS of the total cohort, training cohort, and validation cohort. In all cohorts, the median OS was 56 (95% CI 52–63), while it was and 24 (95% CI 22–28) in the moderate-risk and high-risk groups, respectively, and no median survival was observed in the low-risk group.

### Effects of Radiotherapy on the Survival Benefits in Different Stratifications

**Figure 7:**
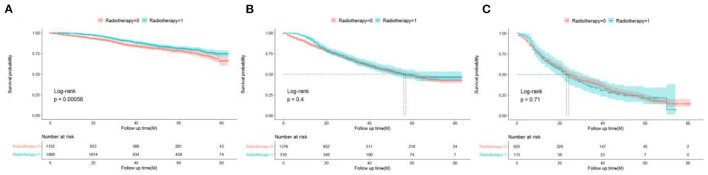
Survival benefits of radiotherapy in low- **(A)**, medium- **(B)**, and high-risk **(C)** groups in elderly patients with TNBC.

To further evaluate the survival benefit of radiotherapy, Kaplan–Meier curves were plotted in the low-, medium-, and high-risk groups (Figures 7A–C). The results showed that radiotherapy prolonged prognosis in the low-risk group (*P* = 0.00056) but did not significantly improve prognosis in the medium-risk group (*P* = 0.4) and high-risk group (*P* = 0.71).

### Construction of Web App for Easy Access of Nomogram

The web app used in this study can be accessed at https://xiaozhuliu.shinyapps.io/dynnomapp/ to assist researchers and clinicians by making it convenient for them to calculate the survival probability of patients.

## Discussion

In this study, a total of 5,677 elderly women were included. Univariate and multivariate Cox analyses determined eight demographic and clinical characteristics, namely, age, race, pathological grade, T stage, N stage, chemotherapy status, radiotherapy status, and tumor size. The model was evaluated by the C-index and calibration charts, and the results showed that the model had good differentiation and calibration. Through a DCA, our nomogram was shown to have a better clinical net and a larger threshold probability range in predicting 1-, 3-, and 5-year OS in the training set and validation set compared with the traditional TNM staging. In addition, a risk stratification system was established based on the total score of each patient in the nomogram. Finally, the survival benefits of radiotherapy were analyzed in the categorized risk groups.

Several studies have previously discussed nomograms regarding the prognoses of patients with TNBC, and our study built precisely on these. However, these previous studies excluded elderly people over 80 years of age or did not further analyze the prognoses of elderly patients with TNBC (see Supplementary Table 1 for details) (17–19). Furthermore, studies have shown that the prognoses of elderly women with BC are generally poor (20). Compared with patients with TNBC of younger ages, elderly patients with TNBC have unique physiological characteristics. These characteristics include more comorbidities, less life expectancy, and worse life expectancy. Thus, it was clear that a prediction model for elderly patients with TNBC needs to be developed. To our knowledge, this is the first large-scale and comprehensive retrospective study to develop a nomogram that could predict the outcomes of older patients with TNBC. Our predictive model can be applied to clinical practice to predict the probability of survival for each patient and alert the physician to the expected benefits of different treatments. In addition, the newly established risk stratification system can identify high-risk patients who require additional adjuvant therapy, shorten the follow-up period of the high-risk subgroup, and adjust the treatment plan in time. Meanwhile, the predictors included in the prediction model can be conveniently obtained from clinical practice.

Pathological grade, T stage, N stage, and tumor size are common risk factors in patients with TNBC and their large values indicate a high risk (18, 19, 21). From our nomogram, it was seen that these four factors in elderly patients with TNBC have similar conclusions with those of patients with TNBC overall. A previous study analyzed the prognoses of young and elderly patients with TNBC and found that the prognosis of elderly patients was poorer (22), Our study showed that the older the patient, the higher the nomogram score, and the lower the survival rate, the poorer the prognosis. In addition, studies have shown that the incidence rate of TNBC in African-American women is higher than that of other races in the US (23, 24). Our research found that African-American women have poorer prognosis than white people. This may be related to the lower socioeconomic status of African-American women, wherein they have less access to medical care, higher obesity rates, and more commonly have the basal-like subtype (25). In addition, we found that races from Asia or Pacific Islanders exhibited better prognoses. This may be because they have more opportunities for healthcare.

Chemotherapy is currently the only systemic treatment to improve TNBC outcomes, as the response of TNBC to neoadjuvant chemotherapy is relatively good (26, 27). Our study found that patients with adjuvant chemotherapy had better prognoses than those without chemotherapy (HR: 0.625). Recently, two large retrospective studies showed that adjuvant chemotherapy can improve the survival and prognoses of elderly patients with TNBC. In particular, a study by Slavica used the large cancer database of Sweden for their analysis and found that the 5-year OS of patients with adjuvant chemotherapy was 12% higher than that of patients who did not participate in chemotherapy through the propensity matching score method. Using the US National Cancer Database, another study by Jennifer also found that the 5-year OS of patients with adjuvant chemotherapy were 15% higher than that of patients who did not participate in chemotherapy (28, 29). Recently, the object of several controversial reports focused on the value of adjuvant radiotherapy for TNBC. Patients with this triple-negative disease from the Danish Breast Cancer Cooperative Group 82b and 82c trials, who had either T3–4 tumors and/or positive lymph nodes, showed no survival benefit for post-mastectomy radiation (30). However, the power of the analysis was limited by the small number of patients with TNBC (only 152). On the other hand, significant improvements were observed in recurrence-free and OS rates compared with mastectomy alone in women with stage I and stage II TNBC after post-mastectomy radiation therapy according to a prospective randomized controlled multicenter trial in China (31). Thus, our study showed for the first time that adjuvant radiotherapy is beneficial for low-risk elderly patients with TNBC (*P* = 0.00056) and shows no benefits between moderate- and high-risk groups (*P* = 0.4 and *P* = 0.71), which can offer guidelines for clinicians to increase the remaining time of their patients.

This study had some limitations. First, only 12 variables in our study were included in this study because SEER does not include all variables. For instance, some important variables such as chemotherapy regimens and detection during surgery were not included. Second, we only involved patients with TNBC that had a histology of IDC and ILC due to the limited number of patients, while other types of TNBC were not included. Third, selection bias may have been present, as this study was a retrospective cohort study that only included patients with complete information on relevant variables. Fourth, the primary population in this study mostly included Americans. Thus, whether the findings are applicable to other populations needs to be further validated in prospective clinical pilot studies.

## Conclusion

This study constructed the first practical nomogram and online web app that can accurately and objectively predict the individualized long-term OS of elderly patients with TNBC based on the clinical risk factors identified from the SEER database. Moreover, the nomogram performed well and had high reliability and accuracy according to the results of the validation cohort. As the first nomogram with an internal validation based on a large series, we believe that the well-established nomogram and the important findings from our study could guide follow-up management strategies for elderly patients with TNBC and help clinicians improve individual treatment.

## Data Availability Statement

The datasets presented in this study can be found in online repositories. The names of the repository/repositories and accession number(s) can be found below: The datasets analyzed for this study can be found in the SEER database (https://seer.cancer.gov/).

## Ethics Statement

Ethical review and approval was not required for the study on human participants in accordance with the local legislation and institutional requirements. Written informed consent for participation was not required for this study in accordance with the national legislation and the institutional requirements.

## Author Contributions

XL, TX, and SY contributed to the conception and design. XL, MD, and HH analysed the data. XL drafted the manuscript. BZ and JL contributed with a critical revision of the manuscript. All authors have read and approved the final version of the manuscript.

## Funding

This study was supported by Science and Technology Innovation Project of Chongqing Science and Technology Commission (cstc2019shm0031), National Natural Science Foundation of China (No. 81801812).

## Conflict of Interest

The authors declare that the research was conducted in the absence of any commercial or financial relationships that could be construed as a potential conflict of interest.

## Publisher's Note

All claims expressed in this article are solely those of the authors and do not necessarily represent those of their affiliated organizations, or those of the publisher, the editors and the reviewers. Any product that may be evaluated in this article, or claim that may be made by its manufacturer, is not guaranteed or endorsed by the publisher.
