# Supplementary material for: Risk Stratification Model for Predicting the Overall Survival of Elderly Triple-Negative Breast Cancer Patients: A Population-Based Study
Source: Front Med (Lausanne). 2021 Sep 21;8:705515. doi: 10.3389/fmed.2021.705515 (PMC8490672; doi:10.3389/fmed.2021.705515)
Supplement: Supplementary file 1 [file Table_1.DOCX]

**Supplementary TABLE1：**

| PMID | This work | 30210925 | 33344259 | 30510456 |
| --- | --- | --- | --- | --- |
| Factors after multivariate analysis | age, race, grade, TN stage,  chemotherapy status, radiotherapy status, and tumor size at diagnosis | grade, tumor stage, tumor size, regional nodes positive, marital status, experience of radiotherapy or chemotherapy | age, race, tumor size, tumor, primary site, and pathological grade | age at diagnosis, race, tumor size, number of positive lymph nodes, grade, and histological subtype |
| The cohort | Females aged over 70 years old，IDC and ILC | Females aged between 20-70 years old，IDC | Females of all ages，IDC | Females aged over 20 years old ，TNBC |
| c-index | 0.750  (95%CI0.742-0.768) | 0.763 | 0.689 | OS:0.774 BCSS:0.792 |
| The Outcome | OS | OS | Lymph node metastasis | OS,BCSS |
| The number of the study population | 5677 | 14538 | 28966 | 21419 |
| The data source | SEER databases | SEER databases | SEER databases | SEER databases |

The study population of the model(PMID:30210925) is IDC patients aged 20-79 and women over 80 years old is not considered, which is easy to cause deviation. To some extent, this shortcoming has been corrected by our study (This work).

The outcome of the model(PMID:33344259) is lymph node metastasis, while the outcome of this study (This work) is OS.

The study population of the model(PMID:30510456) was TNBC patients aged over 20 years old and it compared the TNBC patients over 60 years old with patients under 60 years old and the result showed that the prognosis of patients over 60 years old was worse. However, due to the particularity of elderly TNBC patients, no further discussion has been conducted on this population and our study has corrected this shortcoming.
